# Supplementary material for: Electronic patient-reported outcome measures using mobile health technology in rheumatology: A scoping review
Source: PLoS One. 2021 Jul 22;16(7):e0253615. doi: 10.1371/journal.pone.0253615 (PMC8297791; doi:10.1371/journal.pone.0253615)
Supplement: S1 Appendix — (PDF) [file pone.0253615.s001.pdf]

S1 Appendix. Search strategy (Ovid Medline).

Search concept	Index terms and key words
Patient-reported outcome measures	"Surveys and Questionnaires" [MeSH] OR "Patient Reported Outcome Measures" [MeSH] OR "Self Report" [MeSH] OR "Diagnostic Self Evaluation" [MeSH] OR "Self-Management" [MeSH] OR "Patient Reported Outcome*" OR "Patient Reported Outcome Measure*" OR "ePRO" OR "e-PRO" OR "PROMIS" OR "((remote or self) adj (monitor* or manag*))" OR "(HR-PRO or HRPRO or HRQL or HRQoL or QL or QoL).ti,ab. or quality of life.mp. or (health index* or health indices or health profile*).ti,ab. or health status.mp. or ((patient or self or child or parent or carer or proxy) adj (appraisal* or appraised or report or reported or reporting or rated or rating* or based or assessed or assessment*)).ti,ab. or ((disability or function or functional or functions or subjective or utility or utilities or wellbeing or well being) adj2 (index or indices or instrument or instruments or measure or measures or questionnaire* or profile or profiles or scale or scales or score or scores or status or survey or surveys)).ti,ab."
Rheumatic diseases	"Rheumatic Diseases" [MeSH] OR"Arthritis" [MeSH] OR "Arthritis, Rheumatoid" [MeSH] OR "Osteoarthritis" [MeSH] OR "Arthritis, Reactive" [MeSH] OR "Arthritis, Psoriatic" [MeSH] OR "Arthritis, Juvenile" [MeSH] OR "Arthritis, Gouty" [MeSH] OR "Gout" [MeSH] OR "Lupus Erythematosus, Systemic" [MeSH] OR "Spondylarthropathies" [MeSH] OR "Spondylitis, Ankylosing" [MeSH] OR "Vasculitis" [MeSH] OR "Scleroderma, Systemic" [MeSH] OR "Scleroderma, Limited" [MeSH] OR "Scleroderma, Localized" [MeSH] OR "Scleroderma, Diffuse" [MeSH] OR "Myositis" [MeSH] OR "Dermatomyositis" [MeSH] OR "Polymyositis" [MeSH] OR "Mixed Connective Tissue Disease" [MeSH] OR "Fibromyalgia" [MeSH] OR "Polymyalgia Rheumatica" [MeSH] OR "Sjogren's Syndrome" [MeSH] OR "Rheumatic Disease*" OR "Rheumatologic" OR "Rheumatology" OR “Arthritis” OR "Rheumatoid Arthritis" OR “Osteoarthritis” OR "Reactive Arthritis" OR "Psoriatic Arthritis" OR "Juvenile Idiopathic Arthritis" OR “Gout” OR “Lupus” OR “Spondyloarthropathy” OR “Spondyloarthropathies” OR "Ankylosing Spondylitis" OR “Vasculitis” OR “Scleroderma” OR "Systemic Sclerosis” OR “Myositis” OR “Dermatomyositis” OR “Polymyositis” OR "Mixed Connective Tissue Disease*" OR “Fibromyalgia” OR “Polymyalgia Rheumatica” OR "Sjogren's Syndrome"
Mobile health technology	"Smartphone" [Mesh] OR "Mobile Applications" [Mesh] OR "Computers, Handheld" [Mesh] OR "Cell Phone" [Mesh] OR "Smart Phone*" OR “Smartphone*” OR "Cellular Phone*" OR "Cell Phone*" OR "Cellphone*" OR "Mobile Phone*" OR "Mobile Device*" OR "Handheld Device*" OR "Hand-Held Device*" OR "Handheld Phone*" OR "Hand-Held Phone*" OR “ipad*” OR "Tablet Computer*" OR "Mobile Application*" OR "Mobile App*" OR "m-health" OR “mhealth”
