# Supplementary material for: Electronic patient-reported outcome measures using mobile health technology in rheumatology: A scoping review
Source: PLoS One. 2021 Jul 22;16(7):e0253615. doi: 10.1371/journal.pone.0253615 (PMC8297791; doi:10.1371/journal.pone.0253615)
Supplement: S2 Appendix — (PDF) [file pone.0253615.s002.pdf]

S2 Appendix. Studies included in the scoping review.

1. Allen KD, Coffman CJ, Golightly YM, Stechuchak KM, Keefe FJ. Daily pain variations among patients with hand, hip, and knee osteoarthritis. Osteoarthr Cartil. 2009;17(10):1275–82.
2. Austin L, Sharp CA, van der Veer SN, Machin M, Humphreys J, Mellor P, et al. Providing 'the bigger picture': benefits and feasibility of integrating remote monitoring from smartphones into the electronic health record. Rheumatology. 2020;59(2):367–78.
3. Azevedo R, Bernardes M, Fonseca J, Lima A. Smartphone application for rheumatoid arthritis self-management: cross-sectional study revealed the usefulness, willingness to use and patients' needs. Rheumatol Int. 2015;35(10):1675–85.
4. Beauvais C, Pham T, Montagu G, Vidal C, Dervin G, Baudart P, et al. Development, acceptability and usability of hiboot: A smartphone app to improve medication adherence, safety and self-assessment in patients with inflammatory arthritis. In: Annals of the Rheumatic Diseases. Proceedings of the Annual European Congress of Rheumatology, EULAR; 2019 Jun 12-15; Madrid, Spain. BMJ Publishing Group; 2019. p. 1442–3.
5. Bellamy N, Patel B, Davis T, Dennison S. Electronic data capture using the Womac® NRS 3.1 Index (m-Womac®): A pilot study of repeated independent remote data capture in OA. Inflammopharmacology. 2010;18(3):107–11.
6. Beukenhorst AL, Howells K, Cook L, McBeth J, O'Neill TW, Parkes MJ, et al. Engagement and Participant Experiences With Consumer Smartwatches for Health Research: Longitudinal, Observational Feasibility Study. JMIR mHealth uHealth. 2020;8(1):e14368.
7. Bingham CO, Gaich CL, DeLozier AM, Engstrom KD, Naegeli AN, de Bono S, et al. Use of daily electronic patient-reported outcome (PRO) diaries in randomized controlled trials for rheumatoid arthritis: rationale and implementation. Trials. 2019;20(1):182.
8. Bird P, Griffiths H, Littlejohn G, Youssef P, Joshua F, Nash P, et al. Fatigue, poor health and mood disturbance persist in rheumatoid arthritis and psoriatic arthritis patients despite disease remission: The opal deeper study. In: Arthritis and Rheumatology. Proceedings of the American

College of Rheumatology/Association of Rheumatology Health Professionals Annual Scientific Meeting, ACR/ARHP; 2017 Nov 3-8; San Diego, CA, United States. John Wiley and Sons Inc.; 2017.

9. Bromberg MH, Connelly M, Anthony KK, Gil KM, Schanberg LE. Prospective Mediation Models of Sleep, Pain, and Daily Function in Children With Arthritis Using Ecological Momentary Assessment. Clin J Pain. 2016;32(6):471–7.

10. Bromberg MH, Connelly M, Anthony KK, Gil KM, Schanberg LE. Self-Reported Pain and Disease Symptoms Persist in Juvenile Idiopathic Arthritis Despite Treatment Advances: An Electronic Diary Study. Arthritis Rheumatol. 2014;66(2):462–9.

11. Cai RA, Beste D, Chaplin H, Varakliotis S, Suffield L, Josephs F, et al. Developing and Evaluating JIApp: Acceptability and Usability of a Smartphone App System to Improve Self-Management in Young People With Juvenile Idiopathic Arthritis. JMIR mHealth uHealth. 2017;5(8):e121.

12. Christie A, Dagfinrud H, Dale Ø, Schulz T, Hagen KB. Collection of patient-reported outcomes; -text messages on mobile phones provide valid scores and high response rates. BMC Med Res Methodol. 2014;14:52.

13. Christie A, Dagfinrud H, Mowinckel P, Hagen KB. Variation in fatigue may be poorly explained by pain: results from a longitudinal, exploratory study. Rheumatol Int. 2016;36(2):279–82.

14. Connelly M, Bromberg MH, Anthony KK, Gil KM, Schanberg LE. Use of smartphones to prospectively evaluate predictors and outcomes of caregiver responses to pain in youth with chronic disease. Pain. 2017;158(4):629–36.

15. Crouthamel M, Quattrocchi E, Watts S, Wang S, Berry P, Garcia-Gancedo L, et al. Using a researchkit smartphone app to collect rheumatoid arthritis symptoms from real-world participants: Feasibility study. JMIR mHealth uHealth. 2018;6(9):e177.

16. Curtis JR, Yang S, Clinton C, Chen L, Nowell WB, Yun H, et al. “doctor, a storm is coming and my joints hurt”: Evaluating associations between weather changes and arthritis symptoms. In:

Arthritis and Rheumatology. Proceedings of the American College of Rheumatology/Association of Rheumatology Health Professionals Annual Scientific Meeting, ACR/ARHP; 2018 Oct 19-24; Chicago, IL, United States. John Wiley and Sons Inc.; 2018. p. 1267–8.

17. Elmagboul N, Coburn BW, Foster J, Mudano A, Melnick J, Bergman D, et al. Comparison of an interactive voice response system and smartphone application in the identification of gout flares. Arthritis Res Ther. 2019;21(1):160.

18. Feuchtenberger M, Kleinert S, Schuch F, Spähtling-Mestekemper S, Kuhn C, Welcker M. Patient Self-Management Apps as one Module of an Integrated Tight-control Concept Based on the Examples of the Digital Applications Rheumalive and Axsplalive. In: Annals of the Rheumatic Diseases. Proceedings of the Annual European Congress of Rheumatology, EULAR; 2017 Jun 14-17; Madrid, Spain. BMJ Publishing Group; 2017. p. 1440.

19. Fritz S, Carandang K, Gibson D. Using a mobile app to facilitate patient-doctor discussions to make informed decisions regarding "painsomnia." In: Arthritis and Rheumatology. Proceedings of the American College of Rheumatology/Association of Rheumatology Health Professionals Annual Scientific Meeting, ACR/ARHP; 2018 Oct 19-24; Chicago, IL, United States. John Wiley and Sons Inc.; 2018. p. 3394–5.

20. Gavigan K, Nowell WB, Serna MS, Stark JL, Yassine M, Curtis JR. Barriers to treatment optimization and achievement of patients' goals: perspectives from people living with rheumatoid arthritis enrolled in the ArthritisPower registry. Arthritis Res Ther. 2020;22(1):4.

21. Geuens J, Geurts L, Swinnen TW, Westhovens R, Vanden Abeele V. Mobile Health Features Supporting Self-Management Behavior in Patients With Chronic Arthritis: Mixed-Methods Approach on Patient Preferences. JMIR mHealth uHealth. 2019;7(3):e12535.

22. Gossec L, Cantagrel A, Soubrier M, Berthelot J-M, Joubert J-M, Combe B, et al. An e-health interactive self-assessment website (Sanoia®) in rheumatoid arthritis. A 12-month randomized controlled trial in 320 patients. Jt Bone Spine. 2018;85(6):709–14.

23. Grainger R, Townsley H, Langlotz T, Taylor W. Patient-clinician co-design co-participation in

design of an app for rheumatoid arthritis management via telehealth yields an app with high usability and acceptance. Stud Health Technol Inform. 2017;245:1223.

24. Harbottle V, Bennett J, Duong C, McErlane F, Foster H. Feasibility Of Wearable Technologies In Children And Young People With Juvenile Idiopathic Arthritis. In: Rheumatology. Proceedings of the British Society for Paediatric and Adolescent Rheumatology Annual Conference, BSPAR; 2017 Oct 4-6; Sheffield, United Kingdom. Oxford University Press; 2017. p. vi2.

25. Harris HE, Brannan S, Venters G, McQuillian A, Lovegrove F, Gibson J, et al. Have You Been To Capri? WWW.CAPRI.SCOT.NHS.UK: Early Results of a Clinic for Arthritis Patients in Remission on the INTERNET. In: Rheumatology. Proceedings of the British Society for Rheumatology and British Health Professionals in Rheumatology Annual Meeting; 2012 May 1-3; Glasgow, United Kingdom. Oxford University Press; 2012. p. iii45.

26. Heiberg T, Kvien TK, Dale Ø, Mowinckel P, Aanerud GJ, Songe-Møller AB, et al. Daily health status registration (patient diary) in patients with rheumatoid arthritis: A comparison between personal digital assistant and paper-pencil format. Arthritis Rheum. 2007;57(3):454–60.

27. Huang J, Xie T, Shu Q, Yang J, Wang Y, Wang H, et al. Influential Factors in Promoting Treat-To-Target for Systemic Lupus Erythematosus Via Empowering Patients: a Cohort Study From China By Smart System of Disease Management (SSDM). In: Annals of the Rheumatic Diseases. Proceedings of the Annual European Congress of Rheumatology, EULAR; 2019 Jun 12-15; Madrid, Spain. BMJ Publishing Group; 2019. p. 205.

28. Ji XJ, Sun K, Hu ZY, Zhang Y, Ma YP, Sun Z, et al. [Comparison of clinical manifestations according to HLA-B(27) genotype in ankylosing spondylitis patients: real-world evidence from smart management system for spondyloarthritis]. Zhonghua Nei Ke Za Zhi. 2018;57(3):179–84.

29. Kampling C, Chehab G, Acar H, Becker A, Schneider M. Use, usability and feasibility of a mapp for patients with rheumatoid arthritis-first results. In: Arthritis and Rheumatology. Proceedings of the American College of Rheumatology/Association of Rheumatology Health Professionals Annual Scientific Meeting, ACR/ARHP; 2016 Nov 11-16; Washington, DC, United States. John

Wiley and Sons Inc.; 2016. p. 647–8.

30. Kearey P, Popple AE, Warren J, Davis T, Bellamy N. Improvement in condition-specific and generic quality of life outcomes in patients with knee osteoarthritis following single-injection Synvisc: results from the LOBRAS study. Curr Med Res Opin. 2017;33(3):409–19.

31. Khurana L, Durand EM, Gary ST, Otero A V., Gerzon MC, Beck J, et al. Subjects with osteoarthritis can easily use a handheld touch screen electronic device to report medication use: Qualitative results from a usability study. Patient Prefer Adherence. 2016;10:2171–9.

32. Klein M, Swart J, Roock S, Willemsen A, Buijsse N, Smink G, et al. Reuma2Go-App Creates a Continuum of Care. In: Pediatric Rheumatology. Proceedings of the 25th European Paediatric Rheumatology Congress, PReS; 2018 Sep 5-8; Lisbon, Portugal. BioMed Central Ltd.; 2018.

33. Kuusalo L, Sokka-Isler T, Kautiainen H, Ekman P, Kauppi MJ, Pirilä L, et al. Automated Text Message–Enhanced Monitoring Versus Routine Monitoring in Early Rheumatoid Arthritis: A Randomized Trial. Arthritis Care Res. 2020;72(3):319–25.

34. Lee J, Park S, Ju JH, Cho JH. Application of a real-time pain monitoring system in Korean fibromyalgia patients: A pilot study. Int J Rheum Dis. 2019;22(5):934–9.

35. Lee RR, Rashid A, Ghio D, Thomson W, Cordingley L. “Seeing Pain Differently”: A Qualitative Investigation Into the Differences and Similarities of Pain and Rheumatology Specialists’ Interpretation of Multidimensional Mobile Health Pain Data From Children and Young People With Juvenile Idiopathic Arthritis. JMIR mHealth uHealth. 2019;7(7):e12952.

36. Lee RR, Shoop-Worrall S, Rashid A, Thomson W, Cordingley L. “Asking Too Much?”: Randomized N-of-1 Trial Exploring Patient Preferences and Measurement Reactivity to Frequent Use of Remote Multidimensional Pain Assessments in Children and Young People With Juvenile Idiopathic Arthritis. J Med Internet Res. 2020;22(1):e14503.

37. Lee SSS, Xin X, Lee WP, Sim EJ, Tan B, Bien MPG, et al. The feasibility of using SMS as a health survey tool: An exploratory study in patients with rheumatoid arthritis. Int J Med Inform. 2013;82(5):427–34.

38. Li X, Sun H, Wu R, Wei H, Fan W, Zhao C, et al. Correlation between disease activity and mental health in chinese patients with rheumatoid arthritis -assessment with smart system of disease management (SSDM) mobiles tools. In: Annals of the Rheumatic Diseases. Proceedings of the Annual European Congress of Rheumatology, EULAR; 2017 Jun 14-17; Madrid, Spain. BMJ Publishing Group; 2017. p. 547.

39. Liu X, Xiao F, Yang J, Mu R, Wang H, Wei H, et al. Major Clinical Characteristics of Chinese Rheumatoid Arthritis (RA) Patients with Smart System of Disease Management (SSDM) under Treat-To-Target (T2T) Recommendations. In: International Journal of Rheumatic Diseases. Proceedings of the 18th Asia Pacific League of Associations for Rheumatology Congress, APLAR; 2016 Sep 26-29; Shanghai, China. Blackwell Publishing; 2016. p. 211.

40. Manini TM, Mendoza T, Battula M, Davoudi A, Kheirkhahan M, Young ME, et al. Perception of Older Adults Toward Smartwatch Technology for Assessing Pain and Related Patient-Reported Outcomes: Pilot Study. JMIR mHealth uHealth. 2019;7(3):e10044.

41. Mollard E, Michaud K. A Mobile App With Optical Imaging for the Self-Management of Hand Rheumatoid Arthritis: Pilot Study. JMIR mHealth uHealth. 2018;6(10):e12221.

42. Mu R, Yang J, Wang H, Duan X, Dong J, Zhang F, et al. Analysis of joints susceptible to rheumatoid arthritis (RA) and their recovery sequence based on DAS28 and physical function based on HAQ with smart system of disease management (SSDM) in China: A prospective cohort study. In: Annals of the Rheumatic Diseases. Proceedings of the Annual European Congress of Rheumatology of the European League Against Rheumatism, EULAR; 2016 Jun 8-11; London, United Kingdom. BMJ Publishing Group; 2016. p. 998–9.

43. Navarro-Millán I, Zinski A, Shurbaji S, Johnson B, Fraenkel L, Willig J, et al. Perspectives of Rheumatoid Arthritis Patients on Electronic Communication and Patient-Reported Outcome Data Collection: A Qualitative Study. Arthritis Care Res (Hoboken). 2019;71(1):80–7.

44. Nigrovic PA, Beukelman T, Tomlinson G, Feldman BM, Schanberg LE, Kimura Y. Bayesian comparative effectiveness study of four consensus treatment plans for initial management of

systemic juvenile idiopathic arthritis: FiRst-Line Options for Systemic juvenile idiopathic arthritis Treatment (FROST). Clin Trials. 2018;15(3):268–77.

45. Nishiguchi S, Ito H, Yamada M, Yoshitomi H, Furu M, Ito T, et al. Self-assessment of Rheumatoid arthritis disease activity using a smartphone application: Development and 3-month feasibility study. Methods Inf Med. 2016;55(1):65–9.

46. Nishiguchi S, Ito H, Yamada M, Yoshitomi H, Furu M, Ito T, et al. Self-assessment tool of disease activity of rheumatoid arthritis by using a smartphone application. Telemed e-Health. 2014;20(3):235–40.

47. Nowell W, Yun H, Willig J, Beaumont J, Johnson B, Ginsberg S, et al. What factors relate to patients contributing longitudinal data using smartphone technology? findings from ra patients participating in arthritispower registry. In: Annals of the Rheumatic Diseases. Proceedings of the Annual European Congress of Rheumatology, EULAR; 2017 Jun 14-17; Madrid, Spain. BMJ Publishing Group; 2017. p. 448–9.

48. Ogdie A, George M, Bush K, Patel M, Nowell WB, Baker J. The patient experience: A process evaluation of a pilot pragmatic using remote monitoring of symptoms. In: Annals of the Rheumatic Diseases. Proceedings of the Annual European Congress of Rheumatology, EULAR; 2019 Jun 12-15; Madrid, Spain. BMJ Publishing Group; 2019. p. 2103.

49. Okifuji A, Bradshaw DH, Donaldson GW, Turk DC. Sequential Analyses of Daily Symptoms in Women With Fibromyalgia Syndrome. J Pain. 2011;12(1):84–93.

50. Perraudin CGM, Illiano VP, Calvo F, O'Hare E, Donnelly SC, Mullan RH, et al. Observational Study of a Wearable Sensor and Smartphone Application Supporting Unsupervised Exercises to Assess Pain and Stiffness. Digit Biomarkers. 2018;2(3):106–25.

51. Reade S, Spencer K, Sergeant JC, Sperrin M, Schultz DM, Ainsworth J, et al. Cloudy with a Chance of Pain: Engagement and Subsequent Attrition of Daily Data Entry in a Smartphone Pilot Study Tracking Weather, Disease Severity, and Physical Activity in Patients With Rheumatoid Arthritis. JMIR mHealth uHealth. 2017;5(3):e37.

52. Richter JG, Kampling C, Chehab G, Acar H, Becker A. Patients with rheumatoid arthritis in Germany: Are they ready for ehealth via mobile medical applications? In: Arthritis and Rheumatology. Proceedings of the American College of Rheumatology/Association of Rheumatology Health Professionals Annual Scientific Meeting, ACR/ARHP; 2016 Nov 11-16; Washington, DC, United States. John Wiley and Sons Inc.; 2016. p. 105–6.

53. Rickmann J, Schiøttz-Christensen B, Dam A. Rheumabuddy, A Smartphone APP, Used to Empower Patients with Rheumatoid Arthritis. In: Annals of the Rheumatic Diseases. Proceedings of the Annual European Congress of Rheumatology of the European League Against Rheumatism, EULAR; 2015 Jun 10-13; Rome, Italy. BMJ Publishing Group; 2015. p. 192.

54. Rong M, Xiao F, Li C, Guan J, Shen Y, Zhao D, et al. Feasibility and Influential Factors in Performing Self-Evaluation of DAS28 with Smart System of Disease Management (SSDM) By RA Patient in China. In: Arthritis and Rheumatology. Proceedings of the American College of Rheumatology/Association of Rheumatology Health Professionals Annual Scientific Meeting, ACR/ARHP; 2015 Nov 6-11; San Francisco, CA, United States. John Wiley and Sons Inc.; 2015.

55. Schnitzer TJ, Yeasted R, Huang L, Duffecy J, Begale M, Apkarian A V. Osteoarthritis pain: Variability and clinical correlations. In: Arthritis and Rheumatism. Proceedings of the American College of Rheumatology/Association of Rheumatology Health Professionals Annual Scientific Meeting, ACR/ARHP; 2013 Oct 25-30; San Diego, CA, United States. John Wiley and Sons Inc.; 2013. p. S907–8.

56. Sikorska-Siudek K, Przygodzka M, Bojanowski S, Radomski R. Mobile Application for Patients with Rheumatoid Arthritis (RA) as a Supporting Tool for Disease Activity Monitoring: Its Usability and Interoperability. In: Annals of the Rheumatic Diseases. Proceedings of the Annual European Congress of Rheumatology of the European League Against Rheumatism, EULAR; 2015 Jun 10-13; Rome, Italy. BMJ Publishing Group; 2015. p. 986.

57. Solomon D, Lu F, Xu C, Colls J, Suh DH, Murray M, et al. Patient adherence with a smartphone app for disease monitoring in rheumatoid arthritis. In: Annals of the Rheumatic Diseases.

Proceedings of the Annual European Congress of Rheumatology, EULAR; 2019 Jun 12-15; Madrid, Spain. BMJ Publishing Group; 2019. p. 350–1.

58. Stinson JN, Lalloo C, Harris L, Isaac L, Campbell F, Brown S, et al. iCanCope with Pain ™: User-Centred Design of a Web- and Mobile-Based Self-Management Program for Youth with Chronic Pain Based on Identified Health Care Needs. Pain Res Manag. 2014;19(5):257–65.

59. Tapping P, Rashid A, Thomson W, Ghio D, Cordingley L, Calam R, et al. "This Feeling!": Can a New Ipad App Help Children with Juvenile Idiopathic Arthritis Communicate their Pain Experiences? Feasibility, Usability and Acceptability. In: Annals of the Rheumatic Diseases. Proceedings of the Annual European Congress of Rheumatology of the European League Against Rheumatism, EULAR; 2015 Jun 10-13; Rome, Italy. BMJ Publishing Group; 2015. p. 128.

60. Walker UA, Mueller RB, Jaeger VK, Theiler R, Forster A, Dufner P, et al. Disease activity dynamics in rheumatoid arthritis: patients' self-assessment of disease activity via WebApp. Rheumatology. 2017;56(10):1707–12.

61. Wang G, Yang J, Duan X, Wu Z, Huang J, Ru J, et al. Identification of major clinical characteristics of Chinese SLE patients and linear correlations among SLEDAI, SF-36, and HADS-ANXIETY using mobile smart system of disease management (SSDM). In: Lupus Science and Medicine. Proceedings of the 12th International Congress on Systemic Lupus Erythematosus, LUPUS, and the 7th Asian Congress on Autoimmunity, ACA; 2017 Mar 26-29; Melbourne, VIC, Australia. BMJ Publishing Group; 2017. p. A115.

62. Wang H.;Zhang B.;Ma J.;Wu Z.;Ding Y. The role of a smart system of disease management in treat-to-target of rheumatoid arthritis treatment. In: International Journal of Rheumatic Diseases. Proceedings of the 18th Asia Pacific League of Associations for Rheumatology Congress, APLAR; 2016 Sep 26-29; Shanghai, China. Blackwell Publishing; 2016. p. 204.

63. Wang Y, Luo L, Li Q, Wang F, Huang A, Zhang H, et al. Improving clinical outcome and reducing cost for patients with rheumatic diseases via online interaction with rheumatologists based on smart system of disease management (SSDM) mobile tool. In: Annals of the Rheumatic

Diseases. Proceedings of the Annual European Congress of Rheumatology, EULAR; 2019 Jun 12-15; Madrid, Spain. BMJ Publishing Group; 2019. p. 2079.

64. Wang Y, Yang L, Yasong L, Wei H, Wu H, Ru J, et al. Psychological profile in patients with rheumatic diseases in China: A study of HADS self-assessment with smart system of disease management (SSDM). In: Annals of the Rheumatic Diseases. Proceedings of the Annual European Congress of Rheumatology, EULAR; 2019 Jun 12-15; Madrid, Spain. BMJ Publishing Group; 2019. p. 1410.

65. Williams B, Muckian B, Peschken C, Furie R, Massarotti E, Sikirica V, et al. Utility of a mobile phone based application to collect patient reported outcome information from subjects with systemic lupus erythematosus. In: Annals of the Rheumatic Diseases. Proceedings of the Annual European Congress of Rheumatology, EULAR; 2019 Jun 12-15; Madrid, Spain. BMJ Publishing Group; 2019. p. 401.

66. Xue J, Song H, Yang J, Zhang Z, Hongbin L, Zhang J, et al. Pattern and Influential Factors in Promoting Treat-To-Target (T2T) for Follow-Up Ankylosing Spondylitis (As) Patients With a Rheumatologist-Patient Interactive Smart System of Disease Management (Ssdm): a Cohort Study From China. In: Annals of the Rheumatic Diseases. Proceedings of the Annual European Congress of Rheumatology, EULAR; 2019 Jun 12-15; Madrid, Spain. BMJ Publishing Group; 2019. p. 490–1.

67. Yuan SLK, Marques AP. Development of ProFibro — a mobile application to promote self-care in patients with fibromyalgia. Physiotherapy. 2018;104(3):311–7.

68. Yun H, Nowell W, Willig J, Beaumont J, Johnson B, Ginsberg S, et al. What factors relate to patients contributing longitudinal data using smartphone technology? findings from ra patients participating in arthritispower registry. In: Arthritis and Rheumatology. Proceedings of the American College of Rheumatology/Association of Rheumatology Health Professionals Annual Scientific Meeting, ACR/ARHP; 2016 Nov 11-16; Washington, DC, United States. John Wiley and Sons Inc.; 2016. p. 18–9.

69. Yun H, Yang S, Nowell WB, Filby C, Chen L. Methotrexate use and fatigue in rheumatoid arthritis patients: Results from a national patient registry. In: Arthritis and Rheumatology. Proceedings of the American College of Rheumatology/Association of Rheumatology Health Professionals Annual Scientific Meeting, ACR/ARHP; 2017 Nov 3-8; San Diego, CA, United States. John Wiley and Sons Inc.; 2017.

70. Zheng H, Tulu B, Choi W, Franklin P. Using mHealth App to Support Treatment Decision-Making for Knee Arthritis: Patient Perspective. eGEMs (Generating Evid Methods to Improv patient outcomes). 2017;5(2):7.
