# Supplementary material for: Electronic patient-reported outcome measures using mobile health technology in rheumatology: A scoping review
Source: PLoS One. 2021 Jul 22;16(7):e0253615. doi: 10.1371/journal.pone.0253615 (PMC8297791; doi:10.1371/journal.pone.0253615)
Supplement: Attachment — Submitted filename: Response to Reviewers PLOS ONE Yacyshyn.docx [file pone.0253615.s004.docx]

June 1^st^, 2021

**RE: Revision #1:** Electronic patient-reported outcome measures using mobile health technology in rheumatology: A scoping review

Dear Editor,

We would like to thank the editorial committee and reviewers of this manuscript for their thoughtful comments, as well as the opportunity to provide a revised version of our manuscript for consideration of publication. Below, we have carefully responded to each comment to the best of our ability. We have also indicated where changes have been made within the manuscript.

Sincerely,

Jaclyn Shelton, on behalf of all authors

**Reviewer #1:**

1. ***The methods of the literature search seem valid; however, I was a little surprised to find out that for example our paper on automated monitoring of early RA using SMS messages published online 11 February 2019 was included as a conference abstract (***[***https://doi.org/10.1002/acr.23846***](https://doi.org/10.1002/acr.23846)***) and not as a full paper. Therefore, I suggest that the authors confirm that the conference abstracts in the reference list actually have been published only as abstracts by March 2020.***

Thank you for this suggestion. Two authors (JS, SC) reviewed all conference abstracts included in the analysis. Each author completed an independent search for corresponding journal articles and later met to discuss any discrepancies between findings. In total, we identified 16 conference proceedings that had corresponding journal articles, published in the time frame of our study, that were not included in our analysis. The article, “Automated Text Message–Enhanced Monitoring Versus Routine Monitoring in Early Rheumatoid Arthritis: A Randomized Trial”, was not captured by our search strategy as it was not indexed at the time of our search. See below.

- Medline: indexed July 10, 2020
- CINAHL: indexed July 9, 2020
- Embase: indexed December 3, 2020
- PsycINFO: not indexed

The other articles can be explained by three main scenarios:

- Scenario A: Full article was not captured by our search strategy, as it was not indexed at the time of our search or there were major differences in conference proceeding title and journal article title.
- Scenario B: Full article was captured by our search strategy, but omitted in the study screening phase.
- Scenario C: Full article was captured by our search strategy and its related conference abstract was also included in the analysis.

Our revised manuscript now includes 70 articles. We have omitted the 16 conference abstracts and have included their corresponding journal articles. Our PRISMA flowchart has also been adjusted accordingly. The total number can be explained by the following formula:

*[76 articles included in original search] – [16 conference proceedings] + [10 new journal articles] = 70*

Detailed explanations of this process were added to our “source of evidence screening and selection” and “search result” sections in our revised manuscript.

Article details are provided in the table below.

| Scenario | Journal article title | Corresponding conference proceeding title |
| --- | --- | --- |
| A | “Providing ‘the bigger picture’: benefits and feasibility of integrating remote monitoring from smartphones into the electronic health record” | Patients' experiences of using a smartphone app for remote monitoring of rheumatoid arthritis, integrated into the electronic medical record, and its impact on consultations |
|  |  | Sharing the burden of rheumatoid arthritis through remote monitoring of rheumatoid arthritis (REMORA): Implications for patients and clinicians |
|  |  | Using smartphones to improve remote monitoring of rheumatoid arthritis: Completeness of patients' symptom reports |
|  | Comparison of an interactive voice response system and smartphone application in the identification of gout flares | Comparison of an interactive voice response (IVR) to smart phone app to determine patient preference for reporting gout flares |
|  | An e-health interactive self-assessment website (Sanoia ®) in rheumatoid arthritis. A 12-month randomized controlled trial in 320 patients | Are rheumatoid arthritis patients willing to use an e-health interactive self-assessment website? analyses of 159 patients from a randomised controlled trial over 12 months |
|  |  | Access to an active, interactive self-assessment e-health platform improves patient-physician communication in rheumatoid arthritis: Results of a randomized controlled trial including 320 patients over 1 year |
|  | Patient-Clinician Co-Design Co-Participation in Design of an App for Rheumatoid Arthritis Management via Telehealth Yields an App with High Usability and Acceptance | Patient-clinician co-participation in design of an APP for RA management via telehealth yields an APP with high usability and acceptance |
|  | Automated Text Message–Enhanced Monitoring Versus Routine Monitoring in Early Rheumatoid Arthritis: A Randomized Trial | Automated cell-phone monitoring of disease activity and medication problems in early rheumatoid arthritis |
|  | Perspectives of Rheumatoid Arthritis Patients on Electronic Communication and Patient Reported Outcome Data Collection: A Qualitative Study | Attitudes and barriers of using electronic data collection to track disease activity outside of clinical settings among patients with rheumatoid arthritis |
|  | iCanCope with Pain™: User-centred design of a web- and mobile-based self-management program for youth with chronic pain based on identified health care needs | iCan cope TM: User-centred design and development of a smartphone app to support self-management for youth with arthritis pain |
|  | Comparison of clinical manifestations according to HLA-B(27) genotype in ankylosing spondylitis patients: real-world evidence from smart management system for spondyloarthritis | Clinical characteristics of 897 chinese ankylosing spondylitis/spondyloarthritis real world cohort: Data from a chinese cohort with smart management system for spondyloarthritis (SMSP) |
| B | Improvement in condition-specific and generic quality of life outcomes in patients with knee osteoarthritis following single-injection Synvisc: results from the LOBRAS study | Clinical benchmarking in knee OA following viscosupplementation with synvisc-one |
|  | Bayesian comparative effectiveness study of four consensus treatment plans for initial management of systemic juvenile idiopathic arthritis: FiRst-Line Options for Systemic juvenile idiopathic arthritis Treatment (FROST) | Characteristics of patients enrolled in the first line options for systemic JIA treatment (FROST) consensus treatment plan study |
| C | Using a ResearchKit Smartphone App to Collect Rheumatoid Arthritis Symptoms From Real-World Participants: Feasibility Study. | Patient rheumatoid arthritis data from the real world (PARADE) study: Preliminary results from an apple researchkitTM mobile app-based real world study in the United States |
|  | Cloudy with a Chance of Pain: Engagement and Subsequent Attrition of Daily Data Entry in a Smartphone Pilot Study Tracking Weather, Disease Severity, and Physical Activity in Patients With Rheumatoid Arthritis. | Daily symptom reports in a smartphone-based study 'cloudy with a chance of pain': Patterns of attrition over the first six months |
|  | Disease activity dynamics in rheumatoid arthritis: patients' self-assessment of disease activity via WebApp | Patient's self-monitoring via smartphone: The compass study correlation between patient self-assessment of rheumatoid arthritis disease activity via smartphone technology and physicians' validated scores |

The addition of the above journal articles resulted in minor changes in our results section. However, the major findings/overall conclusions remain the same and we believe these changes have improved the quality of our manuscript.

2. ***In table 1 two very common quality of life measures, SF-36 and EQ-5D are for some reason listed in the section “other” - a construct of Quality of life should be added – QoL is one of the most central PROMs!***

We agree that the PROMs, SF-36 and EQ-5D, would be better categorized under a “quality of life” subheading. We have made changes to table 1 accordingly.

Of note, the ten journal articles added to our study included more detailed explanations of their ePROMs, when compared to their corresponding conference proceeding(s). As a result, we have made minor adjustments to the subheadings and titles of ePROMs used in table 1 and table 2. We believe that this improved the quality of our manuscript as it more specifically identifies which ePROMs were used. It, however, does not change the overall findings or major conclusions.

3. **Concerning table 2 I have also a comment: I would not refer to DAS28 or HAQ as a disease specific measures for Rheumatoid arthritis – both are used widely in different types of inflammatory arthritis, like undifferentiated arthritis, psoriatic arthritis and peripheral spondyloarthropathy.**

Thank you for this suggestion. We have changed the title of the subheading to “inflammatory arthritis”, rather than “rheumatoid arthritis”.

4. **Conclusions are presented clearly. As a general point I would like to comment that certainly ePROMs will be used increasingly in the future instead of papers filled out in the clinic - it remains to be seen how much research will be conducted on this inevitable change in the way of collecting patient reported data (it should be remembered that the number of the commercial health apps is increasing rapidly). For example, in Finland multiple rheumatology clinics use routinely remote monitoring of PROMs like HAQ, pain VAS and fatigue etc. Personally, I think the question remains how often patients are motivated to fill out PROMs and how they adhere to it – would appropriate interval be weekly or quarterly?**

Thank you for this comment. As described in our discussion, the benefits associated with frequent ePROMs may be limited by poor patient compliance and high attrition rates. We agree that future studies should assess not only the feasibility of ePROM use in clinical practice, but specify the optimal frequency of ePROM use to maximize patient engagement. We have included this statement in our discussion.

**Reviewer #2:**

1. **The abstract should state how many papers the search yielded: "A total of 470 records were identified after duplicates were removed."**

Thank you for this suggestion. We have added this statement under the “results” subheading in our abstract.
